# No Differences in Value-Based Decision-Making Due to Use of Oral Contraceptives

**DOI:** 10.3389/fendo.2022.817825

**Published:** 2022-04-22

**Authors:** Carolin A. Lewis, Ann-Christin S. Kimmig, Nils B. Kroemer, Shakoor Pooseh, Michael N. Smolka, Julia Sacher, Birgit Derntl

**Affiliations:** ^1^ Department of Psychiatry and Psychotherapy, Tübingen Center for Mental Health (TüCMH), University of Tuebingen, Tuebingen, Germany; ^2^ Emotion Neuroimaging Lab, Max Planck Institute for Human Cognitive and Brain Sciences, Leipzig, Germany; ^3^ International Max Planck Research School on Neuroscience of Communication: Function, Structure, and Plasticity, Leipzig, Germany; ^4^ International Max Planck Research School for Cognitive and Systems Neuroscience, University of Tuebingen, Tuebingen, Germany; ^5^ Department of Psychiatry, Technische Universität Dresden, Dresden, Germany; ^6^ Freiburg Center for Data Analysis and Modeling, Albert-Ludwigs-Universität Freiburg, Freiburg, Germany; ^7^ Clinic for Cognitive Neurology, University of Leipzig, Leipzig, Germany

**Keywords:** oral contraceptives, ovarian hormones, value-based decision-making, impulsive choice, delay discounting, probability discounting, risk, loss

## Abstract

Fluctuating ovarian hormones have been shown to affect decision-making processes in women. While emerging evidence suggests effects of endogenous ovarian hormones such as estradiol and progesterone on value-based decision-making in women, the impact of exogenous synthetic hormones, as in most oral contraceptives, is not clear. In a between-subjects design, we assessed measures of value-based decision-making in three groups of women aged 18 to 29 years, during (1) active oral contraceptive intake (N = 22), (2) the early follicular phase of the natural menstrual cycle (N = 20), and (3) the periovulatory phase of the natural menstrual cycle (N = 20). Estradiol, progesterone, testosterone, and sex-hormone binding globulin levels were assessed in all groups *via* blood samples. We used a test battery which measured different facets of value-based decision-making: delay discounting, risk-aversion, risk-seeking, and loss aversion. While hormonal levels did show the expected patterns for the three groups, there were no differences in value-based decision-making parameters. Consequently, Bayes factors showed conclusive evidence in support of the null hypothesis. We conclude that women on oral contraceptives show no differences in value-based decision-making compared to the early follicular and periovulatory natural menstrual cycle phases.

## Introduction

Our everyday life is determined by the decisions and choices which we have made or did not make – no matter how big or small. To make these decisions, we often draw on the cognitive process of value-based decision-making. In this complex cognitive process, potential rewards are balanced against their potential costs, i.e., a certain delay or probability of obtaining or losing something. Value-based decision-making comprises different facets, in which the dimensions amount, delay, or probability differ (1). We speak of *delay discounting* if a person is faced with the decision between a smaller, sooner reward and a larger, later reward. Risk-aversion/seeking and loss aversion are captured in *probability discounting* which is a decision between a sooner, small certain reward (or loss) and a later, less certain but larger reward (or loss). Choice behavior is considered more impulsive if a person tends to choose smaller, sooner rewards over larger, later rewards.

Women and men differ in some aspects of value-based decision-making [for review, see Ambrase et al. (2)]. For example, women show bias towards frequent but smaller rewards, while men tend to maximize rewards even if their strategy is not optimal. Women also tend to regret suboptimal changes in their decision-making strategy and thus are more sensitive to information about previous rewards (3–5). Emerging evidence suggests that ovarian hormones, such as estradiol and progesterone, affect value-based decision-making in women (2). Ovarian hormones fluctuate across the menstrual cycle [~ 28 days; Bull et al. (6)]: In the *follicular* phase, both estradiol and progesterone levels are low in the beginning, with estradiol slowly rising and surging before *ovulation* (periovulatory phase, ~ day 14). Following ovulation, estradiol and progesterone rise again in the *luteal* phase, peaking bluntly. It has been shown that women made more impulsive choices in the early follicular phase, i.e., when both estradiol and progesterone were low, while at the same time women were less likely to wait for a higher reward, compared with the periovulatory phase (7). Similarly, women were also less sensitive for immediate rewards with rising estradiol levels, but this effect was mainly driven by women with lower frontal dopamine levels (8). Hence, decision-making processes may be affected by the interaction between ovarian hormones and neurotransmitter systems involved in decision-making – especially the dopaminergic system (9).

While most studies focused on menstrual cycle related effects on decision-making as the menstrual cycle provides a natural experimental model for investigating influences of endogenous ovarian hormones in women, we know only little about possible effects of exogenous ovarian hormones, such as in oral contraceptives (OCs). More than 100 million women worldwide use OCs (10), as OC-use provides an effective option for contraception as well as for managing cycle-related physiological symptoms. While the physiological side effects of OC-use are relatively well understood (e.g., cardiovascular risk), only little research has been dedicated to the effects of OCs on behavior, brain function or their association with psychopathology [but see (11–14)]. Steep delay discounting, risk-seeking and insensitivity to loss characterize mental disorders such as attention-deficit hyperactivity disorder (15), bipolar disorders (16), or substance use disorders (17). To give but one example, substance use disorders are two times more prevalent in men than in women (18), but women show more severe illness courses [for review see Becker (19)]. In women, drug use escalates more quickly and shows patterns of bingeing more often; moreover, women have poorer outcomes regarding quitting and treatment (20, 21). Evidence from rodent and human studies suggests that effects of ovarian hormones on underlying mechanisms of decision-making contribute to these differences (as reviewed by 2): Women have higher ratings of craving and show greater subjective responses to drug stimuli in the follicular phase compared with the luteal phase. However, a recent review of the relationship between OC-use and smoking-related symptoms found only mixed results, e.g., for craving, and could not report about any published data on OC-use and smoking cessation outcomes (22). Given the fact that one out of four smokers use OCs and that OC-use is related to increased nicotine metabolism (22), further research is needed to explore hormonal treatment developments and, more specifically, to investigate potential benefit/harm and secondary effects of OC-intake.

The most widely prescribed OCs contain a synthetic estrogen (ethinyl estradiol) and a synthetic progesterone (progestin) (10). These combined formulations prevent pregnancies by inhibiting ovulation because endogenous estradiol and progesterone fluctuations are suppressed. While endogenous estradiol and progesterone levels are constantly low in OC-users (23, 24), exogenous hormone levels are on a steadily high level (25). This substitution with higher-affinity, synthetic hormones has been shown to lead to structural brain differences in OC-users compared with naturally cycling women: e.g., OC-users had smaller right putamen volumes (26) as well as lower thickness of the lateral orbitofrontal cortex and the posterior cingulate cortex (27). Especially the lateral orbitofrontal cortex region is essential for the cognitive control of behavior, including response inhibition to stimuli with changing reward value (28). Besides its impact on brain structure, OC-use has also been found to increase resting state functional connectivity in the salience network, central executive network, reward network, as well as in the subcortical limbic network (26), which provides a mechanistic insight for putatively altered value-based decision-making in OC-users.

Overall, results from studies investigating the impact of OCs on value-based decision-making are mixed [for review, see Lewis et al. (29)]. OC-users were more sensitive to monetary rewards and had enhanced blood-oxygen level dependent (BOLD) responses during reward expectation in the anterior insula and inferior prefrontal cortex compared with naturally cycling women (30). Another study found greater neural activation in the amygdala, putamen, and executive frontal areas to food stimuli in OC-users compared with naturally cycling women in the follicular phase, but no differences between OC-users and naturally cycling women in the luteal phase (31). However, these studies were limited by their small sample size [N = 24; (30)] or lack of behavioral outcome measures (31). Two other studies found blunted reward responses in OC-users compared with naturally cycling women: Scheele et al. (32) reported enhanced attractiveness ratings of the partner’s face together with increased BOLD responses in nucleus accumbens and ventral tegmental area in naturally cycling women after oxytocin administration, but not in OC-users. Jakob et al. (33) found that only naturally cycling women showed a significant effect of polymorphisms of the dopamine transporter (DAT1-genotype) on reinforcement learning, while OC-using women did not show any such behavioral variations according to DAT1-genotype differences. Especially the latter study provides a first hypothesis about how decision-making processes may be affected by the interaction between ovarian hormones and neurotransmitter systems involved in decision-making, namely the dopaminergic system. Based on these previous studies, we expect OC-users to show differences in value-based decision-making compared with naturally cycling women. However, we cannot hypothesize the direction of this difference, i.e., if OC-users show more or less impulsive decision-making compared with naturally cycling women.

To this end, we investigated value-based decision-making in women using OCs and compared this group with two other groups of naturally cycling women with different hormonal profiles. In this study, three groups of women underwent a value-based decision-making test battery (34), which measured different facets of value-based decision-making: delay discounting, risk-seeking for gains/losses, and loss aversion. The three hormonal profile groups comprised (1) women using OCs (active pill intake, OC group), (2) women in the early follicular phase (days 2-5 of their cycle, fNC group), and (3) women during the periovulatory phase (± 3 days around ovulation, oNC group). Based on the literature reported earlier, we hypothesized (a) less impulsive choices in the fNC group compared with the oNC group, and (b) differences in value-based decision-making between the OC-group and both naturally cycling groups; the direction of this difference, however, remained exploratory.

## Materials and Methods

### Sample Description

A total of 67 healthy female students were recruited from the University of Tübingen and participated in the study. We excluded five participants: three women did not show a luteinizing hormone (LH) surge in the predefined time frame, two women used progestogen-only contraception or recently switched the OC brand. The remaining 62 participants formed three hormonal profile groups, (1) the OC group (n = 22, mean age = 22 ± 2), (2) the fNC group (n = 20, mean age = 22 ± 3), and (3) the oNC group (n = 20, mean age = 24 ± 4). Inclusion criteria were 18-35 years of age, no history of any neurological or mental disorders and no (other) hormonal treatment within the past three months. For the OC group, we included women using monophasic OCs (containing a synthetic estrogen and a synthetic progesterone; an overview of the oral contraceptives and their compounds used by the study participants can be found in **Supplementary Table 2**) for at least six months (mean duration: 3.3 years ± 1.7 years) and measured them during their active pill intake phase (days 2-21). Inclusion criteria for the fNC and oNC groups were an average cycle length of 21-35 days and no hormonal contraception for the past six months. We tested women in the oNC group during their fertile period, i.e., ± 3 days around the detection of the LH peak (predicting ovulation within 2 days, using NADAL hLH ovulation strips, nal von minden GmbH, Moers/Germany). Women in the oNC group reported the first day of bleeding after the measurement to confirm the test results. We measured women in the fNC group on days 2-5 of their menstruation. All women were comparable in age, verbal intelligence, and executive functioning. **Table 1** shows all sociodemographic and neuropsychological characteristics as well as the serum hormone profiles.

**Table 1:** Sample description (mean and standard deviation) and hormone profiles per hormonal profile group.

Demographic information and questionnaires	OC	fNC	oNC	*p*-value
N	22	20	20	
Age (years)	22 (2)	22 (3)	24 (4)	.208
Impulsiveness (BIS-15)	29.0 (6.4)	28.7 (5.1)	33.0 (7.0)	**.058** ^†^
State anxiety (STAI)	34.9 (9.6)	33.2 (4.8)	33.1 (7.0)	.99
Positive mood (PANAS)	31.9 (6.0)	31.2 (6.2)	30.3 (7.2)	.72
Negative mood (PANAS)	13.8 (4.2)	12.9 (4.0)	12.7 (4.6)	.44
Verbal intelligence (WST)	31.8 (3.2)	32.5 (2.4)	31.7 (3.6)	.69
Executive functioning (TMTB-A in sec)	15.5 (9.5)	15.1 (13.5)	16.2 (12.5)	.96
**Hormone profiles**	**OC**	**fNC**	**oNC**	***p*-value**
Estradiol (pmol/l)	67.0 (30.1)	165.9 (45.8)	516.7 (352.2)	**<.001**, OC = fNC < oNC
Progesterone (nmol/l)	1.3 (0.7)	2.1 (0.9)	6.6 (8.0)	**<.001**, OC = fNC < oNC
Testosterone (nmol/l)	0.8 (0.2)	1.1 (0.3)	1.2 (0.3)	**<.001**, OC < fNC = oNC
SHBG (nmol/l)	182.0 (107.1)	65.1 (33.0)	53.9 (23.2)	**<.001**, OC > fNC = oNC

OC, women using oral contraceptives; fNC, naturally cycling women in the early follicular phase; oNC, naturally cycling women during periovulatory phase; BIS-15, German short version of the Barrat Impulsiveness Scale; STAI, State-Trait Anxiety Inventory; PANAS, Positive and Negative Affect Scale; WST, Wortschatztest; TMTB-A, Trail Making Test; SHBG, sex hormone binding globulin; bold values indicate statistically significant differences; ^†^Marginally significant.

### Experimental Procedure

After we received written informed consent from participants, we checked all inclusion and exclusion criteria and asked for menstrual cycle features, OC intake history, as well as gynecological characteristics (e.g., premenstrual syndrome, pregnancies, endometriosis, polycystic ovary syndrome etc.). The German version of the Structured Clinical Interview [SCID; Wittchen et al. (35)] was used to exclude any history of mental disorder. Neuropsychological tests comprised verbal intelligence [Wortschatztest WST; Schmidt and Metzler (36)] and executive functioning [trail making test TMT; Reitan (37)]. Affective functioning was assessed with the Positive and Negative Affect Scale [PANAS; Watson et al. (38)], state anxiety with the State-Trait Anxiety Inventory [STAI; Laux et al. (39)]. Impulsiveness was assessed with the German short version of the Barrat Impulsiveness Scale [BIS-15; Meule et al. (40)]. Thereafter, participants underwent the value-based decision-making battery (34), as well as two other behavioral tasks (Tübinger Empathy Test and a sexual approach avoidance task; reported in (41). The Ethics committee of the Medical Faculty of Tübingen approved the study.

### Value-Based Decision-Making Battery

The value-based decision-making battery measured different facets of impulsive choice, which were implemented in four tasks: delay discounting, probability discounting for gains, probability discounting for losses, and mixed gambles (34).

Participants repeatedly had to decide for one of two offers, which were presented simultaneously on a computer screen for 5 seconds. Offers were randomly assigned to the left or to the right of the screen and participants had to decide by pressing the respective button. For each trial, the participant’s choice was indicated with a frame before presenting the next offer. The test battery took about 20 minutes. All task choices were hypothetical and participants were not informed about outcomes. Since hypothetical monetary rewards have been shown to produce similar results as real monetary rewards [e.g., (42, 43)], participants were paid a fixed amount of money for compensation after completing the test battery.

The delay discounting (DD) task consisted of 50 trials in which participants had to choose between a smaller, immediate amount of money and a larger, later amount (3-50 €; delays of 3 days, 1 week, 2 weeks, 1 month, 2 months, 6 months or 1 year). This task measured the extent to which individuals discount rewards as a function of delay, where stronger discounting is described by higher k values.

In the probability discounting for gains (PDG) and probability discounting for losses (PDL) tasks, participants had to decide between a small, but sure gain or loss of money and a larger amount of money with changing probabilities (3-50 €, probabilities of 2/3, 1/2, 1/3, 1/4, 1/5; 50 trials respectively). The PDG task measured risk-aversion, described by the preference for sure over probabilistic amounts, which is indicated by higher k values. Higher k values in the PDL task describe a preference for the probabilistic offer over the certain one and therefore captured risk-seeking.

In the mixed gambles (MG) task, participants had to gamble for winning (1-40 €) or losing (5-20 €) money or to reject to gamble over the course of 50 trials. This task measured loss aversion. Higher λ values resulted from participants who tended to reject gambles and therefore weighed losses relatively higher.

The tasks used a trial-by-trial adaptive Bayesian approach, that allows an efficient and precise estimation of the impulsive choice parameters k or λ (34). After each trial, the individual indifference point is estimated based on previous choices and informs the options in the next trial. Additionally, a consistency parameter β was computed for each task. Large values of β describe consistent choices, i.e., a higher probability of choosing the option with a higher value; small values of β represent inconsistent choices. The mathematical modeling and parameter estimation for the four tasks can be found in the **Supplementary Material**, together with the posterior distributions of the estimated parameters k and λ.

The value-based decision-making battery, including instructions, binary choices, outcomes, and the parameter estimation algorithm was implemented using MATLAB, Release 2010a (The MathWorks, Inc., Natick, MA) and Psychotoolbox 3.0.10, based on the Psychophysics Toolbox extensions (44, 45).

### Hormone Sampling and Analysis

Blood levels of estradiol, progesterone, testosterone, and sex hormone binding globulin (SHBG) were assessed to confirm cycle phase and inter-individual differences in sex steroid concentrations. Samples were analyzed using chemiluminescence immunoassays (CLIA; Centaur, Siemens). Measurement units were nmol/l for progesterone, testosterone and SHBG, and pmol/l for estradiol. The analytical sensitivity of the assays is 27.2 pmol/l for estradiol, 0.67 nmol/l for progesterone, 0.09 nmol/l for testosterone, and 1.6 nmol/l for SHBG. For the intra-assay accuracy, the maximum coefficient of variation is 11.1% for estradiol, 12.4% for progesterone, 8.5% for testosterone, and 3.8% for SHBG. The reported overall variation of the assays is 13.3% for estradiol, 12.7% for progesterone, 12.6% for testosterone, and 6.5% for SHBG.

### Data Analysis

Analyses were conducted using R version 3.6.2 (46), using parametric statistical methods with two-tailed significance at *p* <.05. We used log transformations of k, λ, and β to fulfill the assumptions of parametric testing; BIS-15 total scores were centered to the mean. Mean differences between groups in age, questionnaire data, and hormonal profiles were analyzed using univariate ANOVAs. Each task of the battery (DD, PDG, PDL, and MG) was analyzed in separate univariate ANOVAs, with *group* (OC, fNC, and oNC) as between-subjects factor and $\eta_{p}^{2}$ as a measure of effect size. In an exploratory analysis, we also included BIS-15 scores as covariate. We used Pearson’s r to characterize the correlations between k/λ and β. We conducted a sensitivity power analysis using G*Power version 3.1.9.4 (47) to calculate the critical population effect size with 80% power. Our remaining sample (*N* = 62) was sufficiently powered to detect a small to medium effect (*f* ^2^ = 0.21).

## Results

### Demographics and Hormone Concentrations

**Figure 1:**
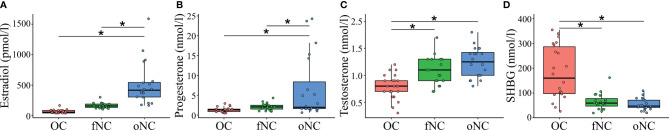
Serum hormone profiles for each hormone profile group, showing **(A)** estradiol in pmol/l and **(B)** progesterone, **(C)** testosterone, and **(D)** sex hormone binding globulin (SHBG) in nmol/l. OC, women using OCs; fNC, women in the early follicular phase; oNC, women during periovulatory phase. Whiskers indicate variability outside the upper and lower quartiles, ‘*’ denote significance levels at *p* <.05.

The hormonal profile groups did not differ in age, mood and anxiety scores, verbal intelligence, and executive functioning (**Table 1**). Impulsiveness differed marginally between groups, as measured with the BIS-15 questionnaire, *F*(2,59) = 2.99, *p* = .058. Hormone concentrations varied as expected across the hormonal phases which were examined (**Table 1** and **Figure 1**): estradiol, *F*(2,59) = 28.08, *p* <.001, progesterone, *F*(2,59) = 7.95, *p* <.001, testosterone, *F*(2,59) = 15.95, *p* <.001, and SHBG, *F*(2,59) = 23.28, *p* <.001 (**Supplementary Table 3** contains single serum hormone profiles for all participants).

### Delay Discounting

**Figure 2:**
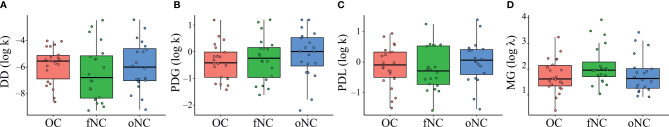
Boxplots showing outcomes for the parameter k of the **(A)** delay discounting (DD), **(B)** probability discounting for gains (PDG), and **(C)** probability discounting for losses (PDL) tasks, and λ of the **(D)** mixed gambles (MG) task. Each dot represents an individual subject. OC, women using OCs; fNC, women in the early follicular phase; oNC, women during periovulatory phase. Whiskers indicate variability outside the upper and lower quartiles.

Running a one-way ANOVA with *group* as between-subjects factor, we found no significant group effect for the DD task, *F*(2,59) = .59, *p* = .560, $\eta_{p}^{2}=.019$ (**Figure 2**). Adding BIS-15 as a covariate did not show any association with parameter k (delay discounting), *F*(3,58) = .42, *p* = .738.

To substantiate the null effect observed in the DD task, a Bayesian analysis approach using the Bayesian information criterion BIC (as described by 48) was applied to allow for the evaluation of the probability of the null hypothesis being true (i.e., that there is no difference between the groups). We provide a detailed description of the approach in the **Supplementary Material**. Bayesian analyses revealed that the probability of the null hypothesis was *p*_BIC_ = .97. According to criteria suggested by Masson [see also (49)], this reflects strong evidence for the null hypothesis (.50-.75 weak,.75-.95 positive,.95-.99 strong, >.99 very strong).

**Figure 3:**
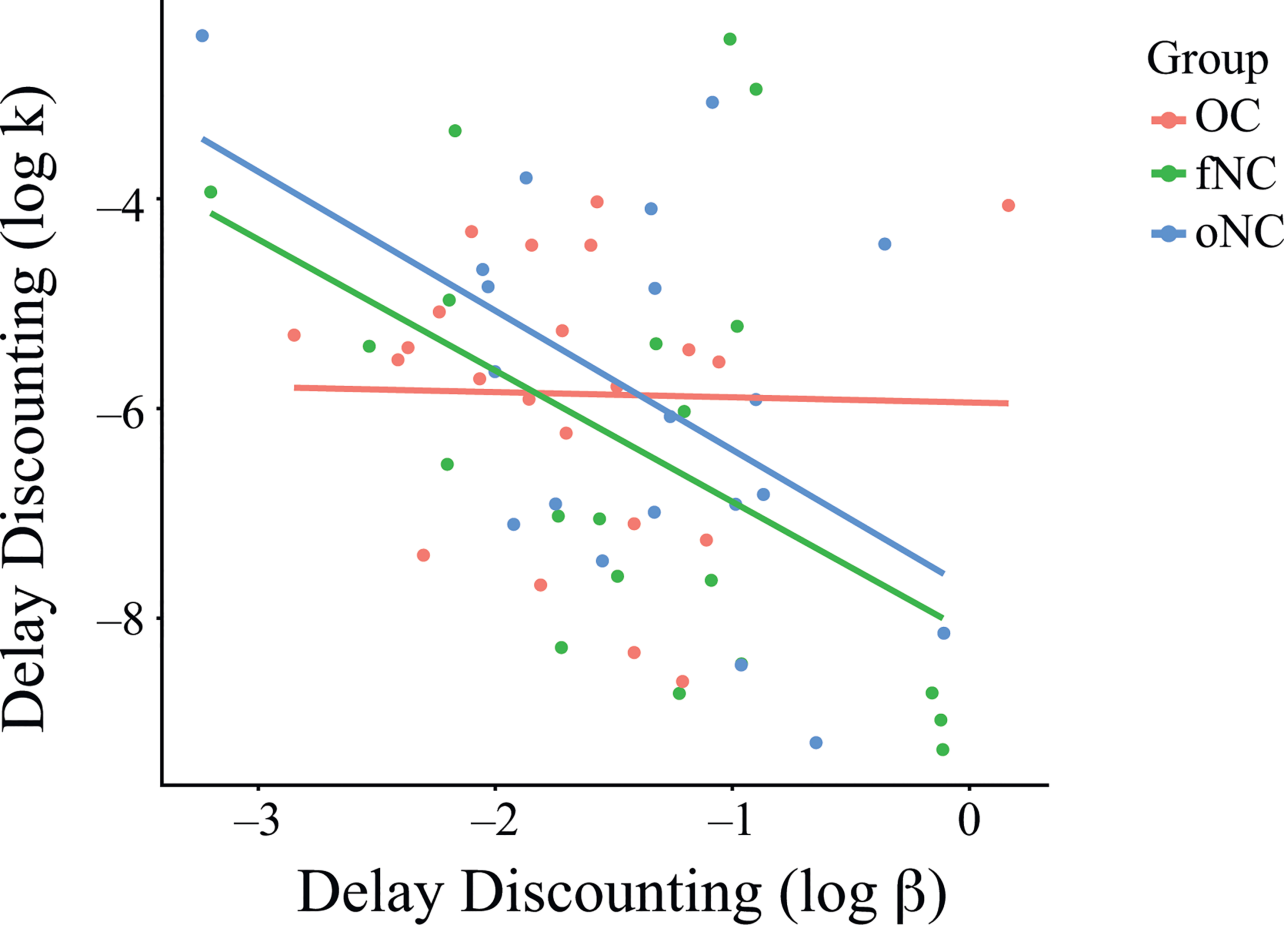
Relationship between the parameters k and β of the delay discounting task per group. Correlation analyses showed significant correlations of k and β for the fNC (*r* = -.48, *p* = .033) and oNC (*r* = -.51, *p* = .021) groups, but not for the OC group (*r* = -.02, *p* = .919; **Figure 3**). However, the correlation coefficients between groups did not differ significantly (fNC vs. OC, *z* = 1.51, *p* = .13; fNC vs. oNC, *z* = 0.12, *p* = .91; oNC vs. OC, *z* = 1.63, *p* = .1; Bonferroni-corrected at *α* = .017). Each dot represents an individual subject. OC, women using OCs; fNC, women in the early follicular phase; oNC, women during periovulatory phase.

Correlation analyses showed significant correlations of k and β for the fNC (*r* = -.48, *p* = .033) and oNC (*r* = -.51, *p* = .021) groups, but not for the OC group (*r* = -.02, *p* = .919; **Figure 3**). In other words, stronger discounting correlated with more inconsistent choices for the naturally cycling groups (fNC and oNC), but not for the OC group. However, the correlation coefficients between groups did not differ significantly (fNC vs. OC, *z* = 1.51, *p* = .13; fNC vs. oNC, *z* = 0.12, *p* = .91; oNC vs. OC, *z* = 1.63, *p* = .1; Bonferroni-corrected at *α* = .017).

### Probability Discounting of Gains

We found no significant differences between groups for the PDG task, using a one-way ANOVA with *group* as between-subjects factor, *F*(2,59) = .55, *p* = .560, $\eta_{p}^{2}=\;.019$ (**Figure 2**). Adding BIS-15 as a covariate did not show any association with parameter k (risk-aversion), *F*(3,58) = 1.17, *p* = .330.

For the PDG task, Bayesian analyses revealed that the probability of the null hypothesis was *p*_BIC_ = .97. This reflects strong evidence for the null hypothesis.

Correlation analyses showed no significant correlations of k and β for any of the groups (OC *r* = -.13, fNC *r* = .05, oNC *r* = -.33; all *p* >.05).

### Probability Discounting of Losses

Running a one-way ANOVA with *group* as between-subjects factor, we found no significant group effect for the PDL task, *F*(2,59) = .67, *p* = .517, $\eta_{p}^{2}=\;.022$ (**Figure 2**). Adding BIS-15 as a covariate did not show any association with parameter k (risk-seeking), *F*(3,58) = .44, *p* = .722.

For the PDL task, Bayesian analyses revealed that the probability of the null hypothesis was *p*_BIC_ = .97. This reflects strong evidence for the null hypothesis.

Correlation analyses showed no significant correlations of k and β for any of the groups, (OC *r* = .15, fNC *r* = -.10, oNC *r* = .14; all *p* >.05).

### Mixed Gambles

**Figure 4:**
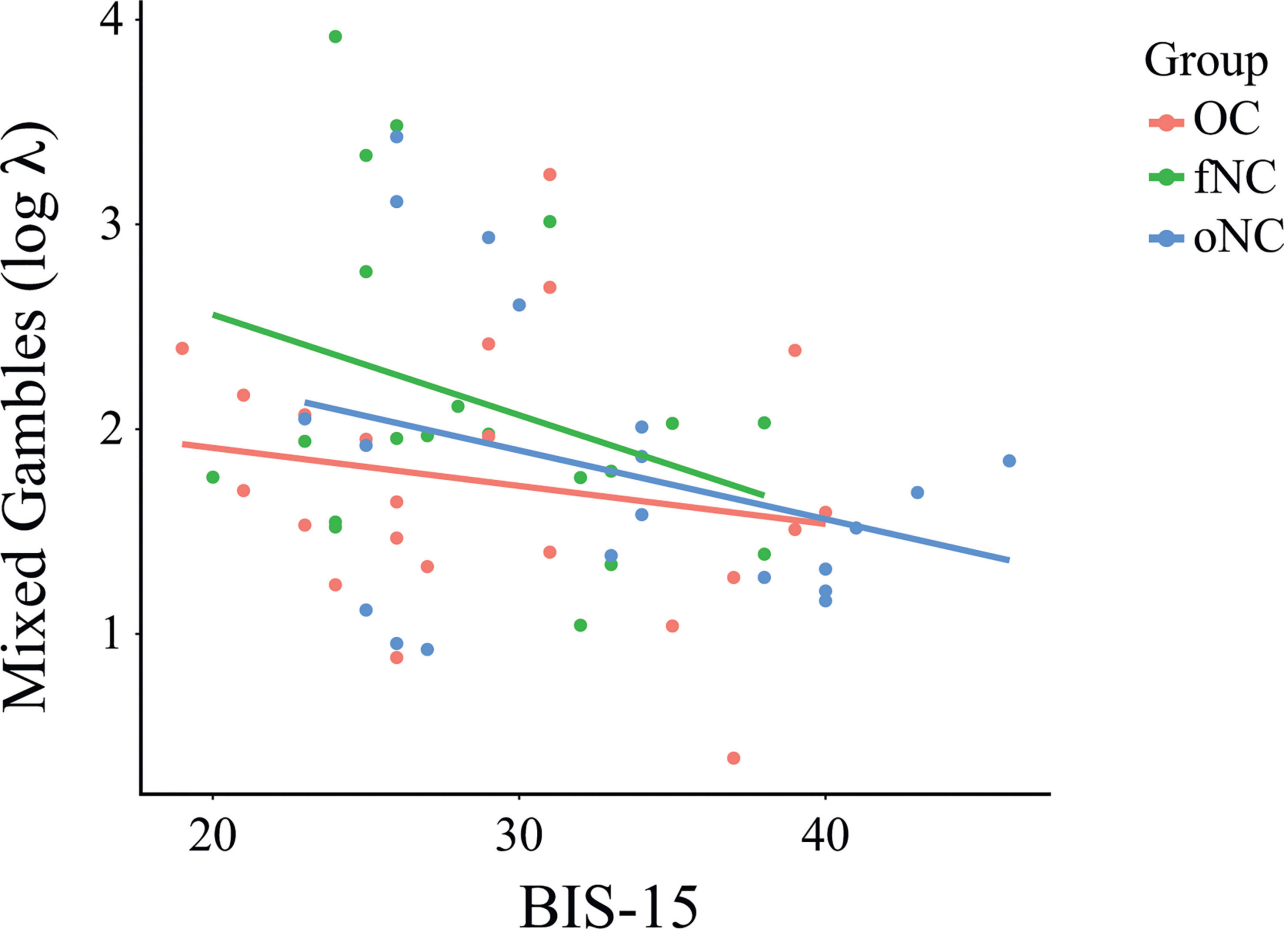
Relationship between the parameter λ (loss aversion) of the mixed gambles task and BIS-15 total score per group. BIS-15 as a covariate was significantly associated with loss aversion for all three groups, *F*(3,58) = 2.87, *p* = .044, $\eta_{p}^{2}=\;.071$ Each dot represents an individual subject. OC, women using OCs; fNC, women in the early follicular phase; oNC, women during periovulatory phase.

We found no significant differences between groups for the MG task, running a one-way ANOVA with *group* as between-subjects factor, *F*(2,59) = 1.83, *p* = .169, $\eta_{p}^{2}=\;.058$ (**Figure 2**). BIS-15 as a covariate was significantly associated with parameter λ (loss aversion) for all three groups, *F*(3,58) = 2.87, *p* = .044, $\eta_{p}^{2}=\;.071$ (**Figure 4**). This means that less impulsive participants tended to reject gambles and therefore weighed losses higher, regardless in which group they were in.

For the MG task, Bayesian analyses revealed that the probability of the null hypothesis was *p*_BIC_ = .91. This reflects positive evidence for the null hypothesis.

Correlation analyses showed no significant correlations of λ and β for any of the groups, (OC *r* = -.33, fNC 22*r* = -.09, oNC *r* = -.12; all *p* >.05).

## Discussion

In the present study, we investigated value-based decision-making in women with different hormonal profiles. We measured the value-based decision-making constructs delay discounting (DD), risk-seeking for gains (PDG) and losses (PDL), and loss aversion (MG). The three groups did not differ in the main outcome parameters k for the DD, PDG, and PDL tasks, and λ for the MG task. We substantiated these null effects using a Bayesian analysis approach, which reflected positive to strong evidence for the null hypothesis, i.e., that there are no differences between groups. The BIS-15 total score as a covariate was not associated with the k parameters of the DD, PDG, and PDL tasks, only with parameter λ (loss aversion) of the MG task. Here, more impulsive participants in all groups tended to reject gambles, which means that they weighed uncertain losses higher than uncertain gains. In a more exploratory fashion, we also ran correlation analyses between k and β to learn more about decision behavior. For the DD task, k and β significantly correlated for the fNC and the oNC groups, but not for the OC group. This means that in naturally cycling women, steeper discounting correlated with more inconsistent choice behavior – but not in women using OCs. Inconsistent choices describe a lower probability of choosing the option with a higher value.

Based on the current literature, we hypothesized (a) less impulsive choices in the fNC group compared with the oNC group, and (b) differences in value-based decision-making between the OC-group and both naturally cycling groups; however, the direction of this difference remained exploratory. Our results did not confirm these hypotheses. One explanation might be the relative scarcity of studies investigating value-based decision-making in different hormonal profile groups. Therefore, formulating straightforward hypotheses might have been premature. Most results so far came from small samples [e.g., Bonenberger et al. (30)], using different tasks, characterizing hormonal profile groups differently [for review, see (29)], and, in general, replication studies are missing [but see Diekhof et al. (50)]. Diekhof et al. (50) replicated results from a within-subjects design in a between-subjects design and showed that avoidance learning capacity is reduced when women were in the high estradiol state of the late follicular phase as compared to the mid luteal phase with more progesterone influence. Although this probabilistic feedback learning task differed in some aspects to the task battery used in our study, the similarity between these tasks lies in maximizing reward by choosing a certain option and, thus, falls within the concept of value-based decision-making. The study by Diekhof et al. (50) not only supports that choice behavior is influenced by hormonal fluctuations, but also confirms the use of between-subjects designs in studies investigating different hormonal states.

Still, in the present study we did not find an effect of different hormonal states on value-based decision-making, especially no difference between the naturally cycling groups and the OC group. One explanation might be a possible hormone-genotype interaction. Jakob et al. (33) investigated how estradiol levels and polymorphisms of the dopamine transporter (DAT1) interact: only naturally cycling women showed a significant effect of DAT1-genotype on reinforcement learning, i.e., a decrease in the ability to avoid punishment with rising estradiol levels in 9RP carriers, while OC-using women did not show any such behavioral variations according to DAT1-genotype differences. This hints at a first hypothesis about how decision-making processes may be affected by the interaction between ovarian hormones and neurotransmitter systems involved in decision-making, namely the dopaminergic system. In the same vein, Jacobs and D'Esposito (51) showed how the interaction between baseline dopamine and estradiol can shape prefrontal cortex dependent working memory performance across the cycle. Here, the effect of estradiol was beneficial or detrimental, depending on the catechol-O-methyltransferase (COMT) genotype, which is involved in metabolizing released dopamine. However, this coupling seems to work differently in women using OCs, leading to no observable variation in behavior (33). The hypothesis of more general differences between naturally cycling women and women using OCs is still uptrend and has been confirmed for several cognitive and behavioral processes [emotion recognition: (13, 52–54); memory performance: (55); fear conditioning and extinction: (56)], however, based on our results, it might not hold true for value-based decision-making.

Evidence increasingly points to considerable effects on brain circuitry and structure following administration of metabolic hormones in form of OC-use [e.g., (26, 27, 57)], however, we do not fully understand the action of OCs on brain and behavior. The present null finding extends the scarce literature on OC-effects on value-based decision-making, especially on the behavioral level. Here, we found no differences between naturally cycling women and women using OCs in making value-based decisions. This result is important for understanding female-specific development, maintenance, and treatment trajectories in mental disorders which are characterized by steep delay discounting, risk-seeking, and insensitivity to loss, as e.g., reported in patients with substance use disorders. It is just as important to know if and how OC-use impacts behavior related to mental health as well as to highlight which behavior is potentially not affected. Still, further research is needed to investigate potential benefit/harm as well as secondary effects of OC-intake on female behavior.

### Limitations

Some limitations have to be noted for the present study. We only used hypothetical monetary rewards. Although hypothetical monetary rewards have been shown to produce similar results as real monetary rewards [e.g., (42, 43)], it would have also been interesting to use real monetary rewards as well as food stimuli. Moreover, we used a relatively new task approach, which has only been used by few studies so far [e.g., (58)]. However, this new approach for adaptive parameter estimation and offer presentation is quick, reliable, and outperforms the most widely used classical approaches.

Also, OC-users in our study had a quite varying mean intake duration of 3.3 years ± 1.7 years. We ruled out a possible impact of these varying intake durations on the results of our study by correlating duration of OC-use with task performance (DD *r* = -.16, PDG *r* = -.12, PDL *r* = .42, MG *r* = -.33; all *p* >.05).

Another limitation is that we only tested young female university students, a group with a presumably very good ability to wait for rewards in the first place. They probably did not differ much in the tested value-based decision-making facets at baseline. One solution would be to use a within-subjects design. However, Diekhof et al. (50) could replicate their results of a within-subjects design in a between-subjects design on avoidance learning capacity and therefore provide first evidence for using between-subjects designs in studies investigating influences of different hormonal states.

Another limitation concerning the study design is that we compared OC-users only with the early follicular and periovulatory phases of naturally cycling women, and not with the luteal phase. Firstly, we aimed at contrasting naturally cycling women, i.e., women with a fluctuating hormonal milieu, with women which do not have hormonal fluctuations, at least over certain period, i.e., during active pill intake. Secondly, we further divided the naturally cycling women in a group with overall low endogenous hormone levels, here the fNC group, and a group with high endogenous estradiol levels, here the oNC group, as we had specific hypotheses based on prior knowledge about the impact of endogenous estradiol on value-based decision-making [e.g., (7, 8)]. The luteal phase of the menstrual cycle shows elevated levels of both estradiol and progesterone, which makes it difficult to disentangle specific effects of either one. To this end, we decided to measure a group with overall low endogenous hormone levels (fNC group) and a group with high endogenous estradiol levels only (oNC group), and compare these groups with women with overall low endogenous hormone levels and high exogenous hormone levels (OC group). Therefore, we could ground our hypotheses about the naturally cycling groups on existing literature on estradiol effects on decision-making and focus on the rather exploratory hypotheses about OC effects in this domain. To substantiate the null findings in our study, we encourage to use larger sample sizes and measure women in a longitudinal design, e.g., a naturally cycling group measured at several time-points during the menstrual cycle in comparison with OC-users measured across a similar time-scale.

### Conclusion

We investigated the impact of different hormonal profiles on the value-based decision-making constructs delay discounting, risk-aversion, risk-seeking, and loss aversion in women. The three groups – early follicular, periovulatory, and OC-using women – did not differ in the main outcome parameters. We underpinned these null effects using a Bayesian analysis approach, i.e., that there are no differences between groups. While more general differences between naturally cycling women and women using OCs have been confirmed for several cognitive and behavioral processes, it might not be the case for value-based decision-making. Understanding the influence of endogenous and exogenous hormones is important in the context of mental disorders with a focus on decision-making deficits and a known sexual dimorphism.

## Data Availability Statement

The raw data supporting the conclusions of this article will be made available by the authors, without undue reservation.

## Ethics Statement

The studies involving human participants were reviewed and approved by Ethics committee of the Medical Faculty of Tübingen. The patients/participants provided their written informed consent to participate in this study.

## Author Contributions

CL, NK, MS, JS, and BD designed the study. SP designed the task. CL and A-CK coordinated the study and acquired the data, which CL analyzed. NK, SP, and MS critically revised the analysis. CL wrote the article, which all authors reviewed. All authors approved the final version to be published and can certify that no other individuals not listed as authors have made substantial contributions to the paper.

## Funding

CL and JS were supported by The Branco Weiss Fellowship – Society in Science, National Association for Research on Schizophrenia and Depression (NARSAD) Young Investigator Grant 25032 from the Brain & Behavior Research Foundation, and by a Minerva Research Group grant from the Max Planck Society (all awarded to JS). CL, A-CK, and BD were supported by the German Research Foundation, DFG (DE2319/9-1, DE2319/2-4). NK was supported by the University of Tübingen, Faculty of Medicine fortune grant #2453-0-0 and the Daimler and Benz Foundation, grant 32-04/19. SP and MS were supported by the German Res earch Foundation (DFG: Deutsche Forschungsgemeinschaft, project numbers 178833530 [SFB 940: Volition and Cognitive Control: Mechanisms, Modulators and Dysfunctions], and 402170461 [TRR 265: Losing and Regaining Control over Drug Intake: Trajectories, Mechanisms, and Interventions])

## Conflict of Interest

The authors declare that the research was conducted in the absence of any commercial or financial relationships that could be construed as a potential conflict of interest.

The reviewer JB declared a past collaboration with one of the authors BD to the handling editor.

## Publisher’s Note

All claims expressed in this article are solely those of the authors and do not necessarily represent those of their affiliated organizations, or those of the publisher, the editors and the reviewers. Any product that may be evaluated in this article, or claim that may be made by its manufacturer, is not guaranteed or endorsed by the publisher.

## References

[1] Green L Myerson J. A Discounting Framework for Choice With Delayed and Probabilistic Rewards. Psychol Bull (2004) 130(5):769–92. doi: 10.1037/0033-2909.130.5.769 PMC1382186 15367080

[2] Ambrase A Lewis CA Barth C Derntl B. Influence of Ovarian Hormones on Value-Based Decision-Making Systems: Contribution to Sexual Dimorphisms in Mental Disorders. Front Neuroendocrinol (2021) 60:100873. doi: 10.1016/j.yfrne.2020.100873 32987043

[3] Byrne KA Worthy DA. Gender Differences in Reward Sensitivity and Information Processing During Decision-Making. J Risk Uncertain (2015) 50(1):55–71. doi: 10.1007/s11166-015-9206-7

[4] Cornwall AC Byrne KA Worthy DA. Gender Differences in Preference for Reward Frequency Versus Reward Magnitude in Decision-Making Under Uncertainty. Pers Individ Dif (2018) 135:40–4. doi: 10.1016/j.paid.2018.06.031 PMC8336713 34354321

[5] Lee TMC Chan CCH Leung AWS Fox PT Gao JH. Sex-Related Differences in Neural Activity During Risk Taking: An fMRI Study. Cereb Cortex (2009) 19(6):1303–12. doi: 10.1093/cercor/bhn172 PMC2677650 18842666

[6] Bull JR Rowland SP Scherwitzl EB Scherwitzl R Danielsson KG Harper J. Real-world Menstrual Cycle Characteristics Of More Than 600,000 Menstrual Cycles. NPJ Digit Med (2019) 2 (1):1–8. doi: 10.1038/s41746-019-0152-7. PMC6710244 31482137

[7] Diekhof EK. Be Quick About it. Endogenous Estradiol Level, Menstrual Cycle Phase and Trait Impulsiveness Predict Impulsive Choice in the Context of Reward Acquisition. Horm Behav (2015) 74:186–93. doi: 10.1016/j.yhbeh.2015.06.001 26092059

[8] Smith CT Sierra Y Oppler SH Boettiger CA. Ovarian Cycle Effects on Immediate Reward Selection Bias in Humans: A Role for Estradiol. J Neurosci (2014) 34(16):5468–76. doi: 10.1523/JNEUROSCI.0014-14.2014 PMC3988406 24741037

[9] Barth C Villringer A Sacher J. Sex Hormones Affect Neurotransmitters and Shape the Adult Female Brain During Hormonal Transition Periods. Front Neurosci (2015) 9:37. doi: 10.3389/fnins.2015.00037 25750611 PMC4335177

[10] United Nations. Trends in Contraceptive Use Worldwide 2015. New York: United Nations, Department of Economic and Social Affairs. (ST/ESA/SER.A/349). (2015).

[11] Bengtsdotter H Lundin C Gemzell Danielsson K Bixo M Baumgart J Marions L. Ongoing or Previous Mental Disorders Predispose to Adverse Mood Reporting During Combined Oral Contraceptive Use. Eur J Contracept Reprod Health Care (2018) 23(1):45–51. doi: 10.1080/13625187.2017.1422239 29323577

[12] Lundin C Danielsson KG Bixo M Moby L Bengtsdotter H Jawad I. Combined Oral Contraceptive Use is Associated With Both Improvement and Worsening of Mood in the Different Phases of the Treatment Cycle-A Double-Blind, Placebo-Controlled Randomized Trial. Psychoneuroendocrinology (2017) 76:135–43. doi: 10.1016/j.psyneuen.2016.11.033 27923181

[13] Scheuringer A Lundin C Derntl B Pletzer B Sundstrom Poromaa I. Use of an Estradiol-Based Combined Oral Contraceptives has No Influence on Attentional Bias or Depressive Symptoms in Healthy Women. Psychoneuroendocrinology (2020) 113:104544. doi: 10.1016/j.psyneuen.2019.104544 31855680

[14] Skovlund C Morch LS Kessing LV Lidegaard O. Association of Hormonal Contraception With Depression. JAMA Psychiatry (2016) 73(11):1154–62. doi: 10.1001/jamapsychiatry.2016.2387 27680324

[15] Jackson JN MacKillop J. Attention-Deficit/Hyperactivity Disorder and Monetary Delay Discounting: A Meta-Analysis of Case-Control Studies. Biol Psychiatry Cognit Neurosci Neuroimaging (2016) 1(4):316–25. doi: 10.1016/j.bpsc.2016.01.007 PMC5049699 27722208

[16] Chandler RA Wakeley J Goodwin GM Rogers RD. Altered Risk-Aversion and Risk-Seeking Behavior in Bipolar Disorder. Biol Psychiatry (2009) 66(9):840–6. doi: 10.1016/j.biopsych.2009.05.011 19615669

[17] Amlung M Vedelago L Acker J Balodis I MacKillop J. Steep Delay Discounting and Addictive Behavior: A Meta-Analysis of Continuous Associations. Addiction (2017) 112(1):51–62. doi: 10.1111/add.13535 PMC5148639 27450931

[18] Grant BF Saha TD Ruan WJ Goldstein RB Chou SP Jung JS. Epidemiology of DSM-5 Drug Use Disorder Results From the National Epidemiologic Survey on Alcohol and Related Conditions-III. JAMA Psychiatry (2016) 73(1):39–47. doi: 10.1001/jamapsychiatry.2015.2132 26580136 PMC5062605

[19] Becker JB. Sex Differences in Addiction. Dialogues Clin Neurosci (2016) 18(4):395–402. doi: 10.31887/DCNS.2016.18.4/jbecker 28179811 PMC5286725

[20] Becker JB Hu M. Sex Differences in Drug Abuse. Front Neuroendocrinol (2008) 29(1):36–47. doi: 10.1016/j.yfrne.2007.07.003 17904621 PMC2235192

[21] Lynch WJ Roth ME Carroll ME. Biological Basis of Sex Differences in Drug Abuse: Preclinical and Clinical Studies. Psychopharmacology (2002) 164(2):121–37. doi: 10.1007/s00213-002-1183-2 12404074

[22] Allen AM Weinberger AH Wetherill RR Howe CL McKee SA. Oral Contraceptives and Cigarette Smoking: A Review of the Literature and Future Directions. Nicotine Tob Res (2019) 21(5):592–601. doi: 10.1093/ntr/ntx258 29165663 PMC6468133

[23] Lobo RA Stanczyk FZ. New Knowledge in the Physiology of Hormonal Contraceptives. Am J Obstet Gynecol (1994) 170:1499–507. doi: 10.1016/S0002-9378(12)91807-4 8178898

[24] Fleischman DS Navarrete CD Fessler DM. Oral Contraceptives Suppress Ovarian Hormone Production. Psychol Sci (2010) 21(5):750–2. doi: 10.1177/0956797610368062 20483856

[25] Collins DC. Sex Hormone Receptor Binding, Progestin Selectivity, and the New Oral Contraceptives. Am J Obstet Gynecol (1994) 170(5):1508–13. doi: 10.1016/S0002-9378(94)05012-X 8178899

[26] Sharma R Smith SA Boukina N Dordari A Mistry A Taylor BC. Use of the Birth Control Pill Affects Stress Reactivity and Brain Structure and Function. Horm Behav (2020) 124:104783. doi: 10.1016/j.yhbeh.2020.104783 32533958

[27] Petersen N Touroutoglou A Andreano JM Cahill L. Oral Contraceptive Pill Use is Associated With Localized Decreases in Cortical Thickness. Hum Brain Mapp (2015) 36(7):2644–54. doi: 10.1002/hbm.22797 PMC4478200 25832993

[28] Elliott R Dolan RJ Frith CD. Dissociable Functions in the Medial and Lateral Orbitofrontal Cortex: Evidence From Human Neuroimaging Studies. Cereb Cortex (2000) 10(3):308–17. doi: 10.1093/cercor/10.3.308 10731225

[29] Lewis CA Kimmig AS Zsido RG Jank A Derntl B Sacher J. Effects of Hormonal Contraceptives on Mood: A Focus on Emotion Recognition and Reactivity, Reward Processing, and Stress Response. Curr Psychiatry Rep (2019) 21(11):115. doi: 10.1007/s11920-019-1095-z 31701260 PMC6838021

[30] Bonenberger M Groschwitz RC Kumpfmueller D Groen G Plener PL Abler B. It's All About Money: Oral Contraception Alters Neural Reward Processing. Neuroreport (2013) 24(17):951–5. doi: 10.1097/WNR.0000000000000024 24136199

[31] Arnoni-Bauer Y Bick A Raz N Imbar T Amos S Agmon O. Is It Me or My Hormones? Neuroendocrine Activation Profiles to Visual Food Stimuli Across the Menstrual Cycle. J Clin Endocrinol Metab (2017) 102(9):3406–14. doi: 10.1210/jc.2016-3921 28911135

[32] Scheele D Plota J Stoffel-Wagner B Maier W Hurlemann R. Hormonal Contraceptives Suppress Oxytocin-Induced Brain Reward Responses to the Partner's Face. Soc Cognit Affect Neurosci (2016) 11(5):767–74. doi: 10.1093/scan/nsv157 PMC4847696 26722017

[33] Jakob K Ehrentreich H Holtfrerich SKC Reimers L Diekhof EK. DAT1-Genotype and Menstrual Cycle, But Not Hormonal Contraception, Modulate Reinforcement Learning: Preliminary Evidence. Front Endocrinol (Lausanne) (2018) 9:60. doi: 10.3389/fendo.2018.00060 29541062 PMC5835510

[34] Pooseh S Bernhardt N Guevara A Huys QJM Smolka MN. Value-Based Decision-Making Battery: A Bayesian Adaptive Approach to Assess Impulsive and Risky Behavior. Behav Res Methods (2018) 50(1):236–49. doi: 10.3758/s13428-017-0866-x 28289888

[35] Wittchen H-U Zaudig M Fydrich T. Strukturiertes Klinisches Interview Für DSM-IV: Achse I Und II. Göttingen: Hogrefe (1997).

[36] Schmidt K-H Metzler P. Wortschatztest (WST). Weinheim: Beltz (1992).

[37] Reitan RM. Trail Making Test. Tucson, AZ: Reitan Neuropsychology Laboratory (1992).

[38] Watson D Clark LA Tellegen A. Development and Validation of Brief Measures of Positive and Negative Affect - The Panas Scales. J Pers Soc Psychol (1988) 54(6):1063–70. doi: 10.1037/0022-3514.54.6.1063 3397865

[39] Laux L Glanzmann P Schaffner P Spielberger CD. Das State-Trait-Angstinventar (STAI): Theoretische Grundlagen Und Handanweisung. Weinheim: Beltz (1981).

[40] Meule A Vögele C Kübler A. Psychometrische Evaluation Der Deutschen Barratt Impulsiveness Scale – Kurzversion (BIS-15). Diagnostica (2011) 57(3):126–33. doi: 10.1026/0012-1924/a000042

[41] Kimmig ACS Wildgruber D Wendel SMU Sundstrom-Poromaa I Derntl B. Friend vs. Foe: Cognitive and Affective Empathy in Women With Different Hormonal States. Front Neurosci (2021) 15:608768. doi: 10.3389/fnins.2021.608768 33762905 PMC7982725

[42] Johnson MW Bickel WK. Within-Subject Comparison of Real and Hypothetical Money Rewards in Delay Discounting. J Exp Anal Behav (2002) 77(2):129–46. doi: 10.1901/jeab.2002.77-129 PMC1284852 11936247

[43] Lagorio CH Madden GJ. Delay Discounting of Real and Hypothetical Rewards III: Steady-State Assessments, Forced-Choice Trials, and All Real Rewards. Behav Processes (2005) 69(2):173–87. doi: 10.1016/j.beproc.2005.02.003 15845306

[44] Brainard DH. The Psychophysics Toolbox. Spat Vis (1997) 10:433–6. doi: 10.1163/156856897X00357 9176952

[45] Pelli DG. The Video Toolbox Software for Visual Psychophysics: Transforming Numbers Into Movies. Spat Vis (1997) 10:437–42. doi: 10.1163/156856897X00366 9176953

[46] R Core Team. R: A Language and Environment for Statistical Computing. Vienna, Austria: R Foundation for Statistical Computing (2019). Available at: https://www.R-project.org/.

[47] Faul F Erdfelder E Lang A-G Buchner A. G*Power 3: A Flexible Statistical Power Analysis Program for the Social, Behavioral, and Biomedical Sciences. Behav Res Methods (2007) 39(2):175–91. doi: 10.3758/BF03193146 17695343

[48] Masson MEJ. A Tutorial on a Practical Bayesian Alternative to Null-Hypothesis Significance Testing. Behav Res Methods (2011) 43(3):679–90. doi: 10.3758/s13428-010-0049-5 21302025

[49] Raftery AE. Bayesian Model Selection in Social Research. Sociol Method (1995) 25:111–63. doi: 10.2307/271063

[50] Diekhof EK Korf S Ott F Schadlich C Holtfrerich SKC. Avoidance Learning Across the Menstrual Cycle: A Conceptual Replication. Front Endocrinol (Lausanne) (2020) 11:231. doi: 10.3389/fendo.2020.00231 32390943 PMC7193994

[51] Jacobs E D'Esposito M. Estrogen Shapes Dopamine-Dependent Cognitive Processes: Implications for Women's Health. J Neurosci (2011) 31(14):5286–93. doi: 10.1523/JNEUROSCI.6394-10.2011 PMC3089976 21471363

[52] Hamstra DA De Rover M De Rijk RH Van der Does W. Oral Contraceptives may Alter the Detection of Emotions in Facial Expressions. Eur Neuropsychopharmacol (2014) 24(11):1855–9. doi: 10.1016/j.euroneuro.2014.08.015 25224104

[53] Pahnke R Mau-Moeller A Junge M Wendt J Weymar M Hamm AO. Oral Contraceptives Impair Complex Emotion Recognition in Healthy Women. Front Neurosci (2019) 12:1041. doi: 10.3389/fnins.2018.01041 30804733 PMC6378414

[54] Radke S Derntl B. Affective Responsiveness is Influenced by Intake of Oral Contraceptives. Eur Neuropsychopharmacol (2016) 26(6):1014–9. doi: 10.1016/j.euroneuro.2016.03.004 27039036

[55] Spalek K Loos E Schicktanz N Hartmann F de Quervain D Stier C. Women Using Hormonal Contraceptives Show Increased Valence Ratings and Memory Performance for Emotional Information. Neuropsychopharmacology (2019) 44(7):1258–64. doi: 10.1038/s41386-019-0362-3 PMC6784990 30836380

[56] Hwang MJ Zsido RG Song HJ Pace-Schott EF Miller KK Lebron-Milad K. Contribution of Estradiol Levels and Hormonal Contraceptives to Sex Differences Within the Fear Network During Fear Conditioning and Extinction. BMC Psychiatry (2015) 15(1):1-12. doi: 10.1186/s12888-015-0673-9 PMC4652367 26581193

[57] Petersen N Kearley NW Ghahremani DG Pochon JB Fry ME Rapkin AJ. Effects of Oral Contraceptive Pills on Mood and Magnetic Resonance Imaging Measures of Prefrontal Cortical Thickness. Mol Psychiatry (2021) 26(3):917–26. doi: 10.1038/s41380-020-00990-2 PMC7914152 33420480

[58] Petzold J Kienast A Lee Y Pooseh S London ED Goschke T. Baseline Impulsivity may Moderate L-DOPA Effects on Value-Based Decision-Making. Sci Rep (2019) 9(1):1–8. doi: 10.1038/s41598-019-42124-x 30948756 PMC6449394

