# Supplementary material for: No Differences in Value-Based Decision-Making Due to Use of Oral Contraceptives
Source: Front Endocrinol (Lausanne). 2022 Apr 22;13:817825. doi: 10.3389/fendo.2022.817825 (PMC9075610; doi:10.3389/fendo.2022.817825)
Supplement: Supplementary file 1 [file DataSheet_1.pdf]

Supplementary Material

1 Mathematical modeling and parameter estimation of the value-based decision-making battery

Delay discounting is described by the function from Mazur (1987):

$$V = \frac{\mathrm{A}}{1 + k_0\,\mathrm{D}} \quad (1)$$

where the subjective value *V* of an outcome of amount *A*, delivered after a delay *D*, declines hyperbolically according to the discounting rate $k_0 > 0$.
With a transformation of the probability to the odds against winning *(1 – p)/p*, the same hyperbolic discounting function is used to describe subjective values of probabilistic outcomes (Rachlin et al., 1991), in our case for the probability discounting of gains and losses tasks:

$$V = \frac{\mathrm{A}}{1 + k_0 \frac{[1 - \mathrm{p}]}{\mathrm{p}}} \quad (2)$$

To estimate a behavioral measure of loss aversion, the equation

$$V = \frac{1}{2}(\mathrm{G} - \lambda\,\mathrm{L}) \quad (3)$$

was used in which loss aversion *λ* is the ratio of the contribution of the loss magnitude *L* to the contribution of the gain magnitude *G* to the participant's decisions (Frydman et al., 2011; Tom et al., 2007).

In the following, we describe the mathematical modeling and parameter estimation algorithm for the case of delay discounting. A Bayesian approach was used to estimate the discounting parameter *k* trial-by-trial by using choices that a person makes between a smaller immediate and a larger delayed reward.
The offers are chosen between r_1 and r_2, measured in a currency unit, and the delays are selected from the set $D = \{d_1, d_2, \ldots, d_7\}$, in days. We assume that the likelihood of choosing between the two offers follows a softmax probability function with an inverse temperature parameter, $\beta_0 > 0$,

$$\boldsymbol{P(a_d|k_0, \beta_0) = 1 - P(a_i|k_0, \beta_0).} \quad \mathbf{(4)}$$

Large values of *β* describe consistent choices, i.e., a higher probability of choosing the option with a higher value; small values of *β* represent inconsistent choices.

The parameters k_0 and β_0 are nonnegative and positively skewed. Parameters were therefore transformed to the natural-logarithmic scale and defined $k = ln(k_0)$ and $\beta = ln(\beta_0)$. The parameters space was discretized over an equally spaced 2-D region, ***R***, with $-8 \leq k \leq 2$ and $-5 \leq \beta \leq$

5. To simplify, the two parameters were assumed to be independent and liberal univariate priors were imposed on the parameters, such that k and β had a Beta and a uniform distribution. By assuming independence, a joint probability distribution $P(k, \beta)$ served as a prior for the Bayesian framework.

Given the prior distribution, the immediate and delayed offers were presented to the participant. The prior was updated after the choice at the first trial, using Bayes's rule:

$$\boldsymbol{P(k,\beta|a) = \frac{1}{Z} - \; P(a|k,\beta)P(k,\beta),} \quad \textbf{(5)}$$

With the joint distribution over the parameters $P(k,\beta)$ and the likelihood $P(a|k,\beta)$ of observing the action a, which results from Eq. 4. For every trial t, the posterior distribution over the parameters, $P(k,\beta|a)$, is updated by multiplying the prior by the likelihood of the participant's action and then becomes the prior for the following trial. In Eq. 5, 1/Z is a normalization factor over the discrete domain $\boldsymbol{R}$. The expected values of k and β served as current parameter estimations $\hat{k}_t$ and $\hat{\beta}_t$ at the end of each trial. By using the current estimates based on the previous choice, the upcoming offers were close to the indifference point, i.e., the probability of choosing the immediate or the delayed offer was equally likely. This approach is considered to provide the most informative data (Lewi et al., 2008; Sebatiani & Wynn, 2000).

In order to present two offers with the same subjective values, the condition

$$\boldsymbol{r_1 \le \frac{r_2}{1+kd} \le r_2 - \delta, \; i = 1, \ldots, m,}$$

holds for all feasible delays, where δ is the minimum difference between the two offers. A feasible delay was chosen randomly, and the following offers were presented such that they differed at least by δ and had the same subjective values according to the current estimate $\hat{k}_t$. After a certain number of trials, N, the resulting $\hat{k}_N$ was considered the estimated parameter. To reduce the probability of participants learning the pattern, random offers were presented throughout the trials.

The same framework is applicable to the concepts of probability discounting of gains/losses and loss aversion.

Please refer to Pooseh et al. (2018) where, through simulations and real data, it has been shown that this method gives similar results compared to standard methods.

1.1 Posterior distributions of the estimated parameters *k* and *λ*

Supplementary Table 1. Posterior distributions of the estimated parameters *k* and *λ* for each participant (posterior means and posterior variances).

Subject ID	mean *k* DD	var *k* DD	mean *k* PDG	var *k* PDG	mean *k* PDL	var *k* PDL	mean *λ* MG	var *λ* MG
fNC_01	-6.03	0.17	-0.61	0.04	-0.56	0.16	1.39	0.05
fNC_02	-5.22	0.08	0.13	0.17	-0.16	0.04	2.77	0.21
fNC_03	-9.25	0.09	0.88	0.12	-1.59	0.15	3.34	0.06
fNC_04	-7.05	0.48	0.25	0.27	-0.52	0.40	1.95	0.08
fNC_06	-5.41	0.85	0.37	0.66	-0.16	0.12	1.55	0.17
fNC_07	-6.53	0.72	-1.31	0.11	0.58	0.23	1.79	0.04
fNC_08	-8.72	0.21	-1.62	0.23	0.56	0.60	1.04	0.01
fNC_09	-8.71	0.09	-0.13	0.05	-0.89	0.27	3.92	0.01
fNC_10	-3.93	1.63	-0.36	0.13	0.52	0.33	1.94	0.42
fNC_11	-3.35	0.28	1.11	0.45	-0.93	0.55	3.01	0.49
fNC_12	-7.64	0.25	-0.94	0.12	-0.23	0.05	2.03	0.002
fNC_13	-5.38	0.29	-0.35	0.32	-0.77	0.09	1.98	0.44
fNC_14	-2.95	0.09	-0.09	0.60	-0.95	0.04	1.97	$9.64\ e^{-5}$
fNC_15	-7.03	0.48	0.12	0.07	-0.25	0.64	1.34	0.01
fNC_16	-4.96	0.62	-1.05	0.01	1.24	0.18	1.76	0.19
fNC_17	-2.48	0.04	0.10	0.12	-0.33	0.53	2.11	0.07
fNC_19	-8.43	0.22	-1.28	0.27	0.57	0.37	1.52	0.26
fNC_20	-8.28	0.38	-0.37	0.10	0.54	0.22	1.77	0.003
fNC_21	-8.97	0.09	1.20	0.003	-0.72	0.34	3.48	0.03
fNC_22	-7.60	0.37	-1.40	0.04	-0.54	0.02	2.03	0.02
OC_01	-4.03	0.27	-0.42	0.05	-0.19	0.11	1.33	0.18
OC_02	-7.40	0.65	-1.22	0.75	-1.30	0.17	1.64	0.36
OC_03	-5.79	0.31	-0.17	0.56	-0.87	0.23	2.42	0.27
OC_04	-6.23	0.33	NA	NA	0.39	0.12	1.47	0.04
OC_06	-5.91	0.51	-0.57	0.02	0.24	0.02	2.07	0.02
OC_08	-4.44	0.14	-0.18	0.67	0.57	0.26	0.88	0.16
OC_09	-5.53	0.59	-0.96	0.62	-0.02	0.01	1.95	0.05
OC_10	-5.08	1.11	-1.42	0.13	-0.31	0.34	1.40	0.03
OC_11	-7.25	0.16	-0.19	0.007	-0.16	0.19	1.04	$9.67\ e^{-4}$
OC_12	-4.06	0.02	0.28	0.34	0.84	0.28	1.96	0.003
OC_13	-5.30	1.70	-1.21	0.66	-0.02	0.45	1.59	0.02
OC_15	-7.10	0.28	-0.44	0.02	-0.37	0.10	2.38	0.05
OC_16	-5.26	0.65	-0.31	0.50	0.33	0.38	1.24	0.005
OC_17	-8.33	0.29	0.58	0.66	-1.51	0.70	3.24	0.16
OC_18	-5.56	0.11	-0.51	0.06	0.32	0.12	1.70	$4.67\ e^{-4}$
OC_19	-5.44	0.17	-0.01	0.07	-0.28	0.02	2.17	0.23
OC_20	-4.44	0.42	0.15	0.28	-0.60	0.26	2.39	0.24
OC_21	-5.72	0.75	0.45	0.16	-1.18	0.09	2.69	0.12
OC_22	-5.42	1.14	-1.01	0.14	-0.54	0.40	1.53	0.002
OC_23	-8.60	0.21	-0.44	0.39	0.60	0.26	0.39	0.04

Subject ID	mean *k* DD	var *k* DD	mean *k* PDG	var *k* PDG	mean *k* PDL	var *k* PDL	mean *λ* MG	var *λ* MG
OC_24	-4.31	0.55	1.19	0.49	0.94	0.12	1.51	0.19
OC_25	-7.68	0.47	-1.27	0.02	0.20	0.11	1.28	0.02
oNC_01	-7.10	0.41	0.58	0.11	-0.53	0.35	1.38	0.05
oNC_02	-5.91	0.09	-0.38	0.07	-0.57	0.10	2.61	0.28
oNC_04	-6.91	0.20	-0.43	0.05	-1.54	0.03	1.69	0.004
oNC_05	-8.45	0.19	0.68	0.50	-0.13	0.45	3.11	0.18
oNC_06	-6.08	0.19	-0.78	0.21	0.42	0.19	1.21	0.04
oNC_07	-4.09	0.20	-0.12	0.37	0.09	0.82	0.95	0.07
oNC_08	-2.44	2.11	0.16	0.11	0.55	0.02	1.28	0.25
oNC_09	-7.45	0.25	0.91	0.56	1.39	0.03	1.92	0.44
oNC_11	-4.67	0.55	0.34	0.52	-0.58	0.12	2.94	0.02
oNC_12	-3.08	0.14	0.16	0.001	0.01	0.17	2.01	0.01
oNC_13	-9.19	0.11	1.20	0.03	0.40	0.25	1.52	0.07
oNC_14	-4.43	0.04	0.51	0.08	0.03	0.39	1.85	0.09
oNC_17	-4.84	0.32	-0.90	0.60	0.37	0.64	0.92	0.18
oNC_18	-6.91	0.41	-1.79	0.04	1.18	0.17	1.32	0.05
oNC_19	-8.14	0.05	0.01	0.33	0.39	0.004	1.12	0.03
oNC_20	-6.82	0.14	1.19	0.68	-1.21	0.13	3.43	0.05
oNC_22	-5.65	0.81	-0.46	0.50	-0.36	0.53	1.87	0.01
oNC_23	-4.85	0.27	0.002	0.66	0.46	0.29	2.05	0.42
oNC_24	-3.80	0.47	-0.77	0.03	-0.07	0.29	1.16	0.07
oNC_25	-6.99	0.25	-2.20	0.01	0.13	0.09	1.58	0.04

2 Bayesian alternative to null-hypothesis significance testing using the Bayesian information criterion

The approach described by Masson (2011) provides evidence regarding which model, i.e., effect absent (null hypothesis) vs. effect present (alternative hypothesis), is more strongly supported by the data. This is in contrast with classic null hypothesis significance testing, which does not allow investigating the degree of support favoring the null hypothesis. This approach uses the Bayesian information criterion (BIC) approximation of Bayesian posterior probabilities introduced by Wagenmakers (2007).

First, we compute ΔBIC, by

$$\Delta BIC = n \ln \frac{SSE_1}{SSE_0} + (k_1 - k_0) \ln(n) \quad (1)$$

where SSE_1 and SSE_0 are the sums of squares for the error terms in the alternative and the null hypothesis models. The term SSE_1/SSE_0 is simply the complement of partial eta-squared η_p^2, an effect size measure which describes the proportion of variability accounted for by the independent variable ($SSE_1/SSE_0 = 1 - \eta_p^2$). The term $k_1 - k_0$ corresponds to the difference in the number of free parameters between the two models, i.e., the degrees of freedom associated with an effect when null and alternative hypotheses are contrasted.

The ΔBIC value can then be used to generate an estimate of the Bayes factor

$$BF = \frac{p_{BIC}(D|H_0)}{p_{BIC}(D|H_1)} = e^{(\Delta BIC)/2} \quad (2)$$

In a final step, the Bayes factor is converted into the posterior probabilities for the two competing hypotheses

$$p_{BIC}(H_0|D) = \frac{\mathrm{BF}}{\mathrm{BF} + 1} \quad (3)$$

Given the fact that posterior probabilities will not always clearly favor one hypothesis over the other, it is recommended to use the convention for labeling the strength of evidence provided by Raftery (1995). Here, p_{BIC} values of .50-.75 are considered as weak, .75-.95 as positive, .95-.99 as strong, and >.99 as very strong.

Please refer to Masson (2011) for example applications of the approach and an Excel worksheet for computing p_{BIC} values.

3 Oral contraceptives used by the study participants

Supplementary Table 2. Overview of the oral contraceptives and their compounds used by the study participants.

Generation	Compounds	Androgen/Antiandrogen	Used by participants
2nd	Ethinylestradiol (20-30 µg), Levonorgestrel, Norethisteron	androgen	11
3rd	Ethinylestradiol (20-30 µg), Desogestrel, Gestoden,	neutral	1
4th	Ethinylestradiol (20-30 µg), Drospirenon, Chlormadinon, Dienogest, Nomegestrol	antiandrogen	10

4 Serum hormone profiles for all participants

Supplementary Table 3 Serum hormone profiles for all participants. Measurement units were pmol/l for estradiol and nmol/l for progesterone, testosterone and serum hormone binding globulin (SHBG).

Subject ID	Age	Estradiol	Progesterone	Testosterone	SHBG
fNC_01	22	137	2.00	1.10	51
fNC_02	20	185	1.20	1.10	70
fNC_03	27	170	4.30	1.30	64
fNC_04	19	117	1.60	1.10	26
fNC_06	23	149	3.40	1.70	95
fNC_07	20	140	2.80	0.90	55
fNC_08	25	204	1.70	1.00	90
fNC_09	24	209	1.00	0.70	94
fNC_10	24	127	0.90	0.80	56
fNC_11	29	121	1.20	0.80	19
fNC_12	20	184	2.20	1.30	35
fNC_13	21	157	2.10	1.30	67
fNC_14	22	207	1.80	1.20	51
fNC_15	25	122	1.00	0.90	73
fNC_16	19	137	2.70	1.70	60
fNC_17	22	302	2.40	1.30	34
fNC_19	24	190	2.40	0.90	106
fNC_20	19	193	2.20	1.40	162
fNC_21	22	161	1.90	0.70	58
fNC_22	19	105	3.10	1.00	35
OC_01	23	91	1.40	0.70	103
OC_02	24	87	2.90	1.00	42
OC_03	23	43	1.10	0.70	300
OC_04	21	43	1.20	0.40	96
OC_06	24	43	1.60	0.60	201
OC_08	20	65	1.20	0.60	93
OC_09	19	87	1.30	0.80	261
OC_10	24	101	2.30	1.10	26
OC_11	19	86	2.20	1.20	119
OC_12	21	47	1.30	0.90	105
OC_13	20	43	1.40	0.80	294
OC_15	22	62	1.50	0.90	208
OC_16	24	43	0.50	0.30	266
OC_17	23	43	2.50	0.80	340
OC_18	20	167	1.30	0.90	102
OC_19	19	43	0.50	1.00	355
OC_20	22	95	1.00	1.10	56
OC_21	23	52	0.60	0.70	177
OC_22	22	53	1.50	0.90	143
OC_23	25	48	0.50	0.80	304

OC_24	25	71	0.80	0.50	84
OC_25	24	60	0.80	0.70	328
oNC_01	23	403	1.70	0.80	44
oNC_02	27	1589	1.60	1.00	44
oNC_04	29	564	2.20	1.50	79
oNC_05	18	307	1.60	1.60	48
oNC_06	19	436	2.90	1.40	93
oNC_07	24	889	4.90	1.40	26
oNC_08	25	206	1.60	1.50	30
oNC_09	25	538	6.40	1.40	46
oNC_11	29	515	1.30	1.30	74
oNC_12	27	1065	1.00	0.90	109
oNC_13	21	167	1.60	1.00	20
oNC_14	22	401	15.30	0.90	58
oNC_17	23	201	1.60	1.40	43
oNC_18	25	302	5.20	1.20	39
oNC_19	29	913	0.60	0.90	79
oNC_20	18	485	18.20	1.50	70
oNC_22	26	370	23.70	1.00	50
oNC_23	22	176	1.20	1.80	48
oNC_24	21	432	14.30	1.10	42
oNC_25	19	374	24.30	1.20	35
